# Poster Session II A334 IMPLEMENTATION EFFECTIVENESS OF MINDFULNESS INTERVENTIONS IN INFLAMMATORY BOWEL DISEASE: A SYSTEMATIC REVIEW

**DOI:** 10.1093/jcag/gwaf042.334

**Published:** 2026-02-13

**Authors:** N Willett, S Lalani, J Jones

**Affiliations:** Division of Digestive Care and Endoscopy, Nova Scotia Health Authority, Halifax, NS, Canada; McGill University Faculty of Medicine and Health Sciences, Montreal, QC, Canada; Division of Digestive Care and Endoscopy, Nova Scotia Health Authority, Halifax, NS, Canada

## Abstract

**Background:**

Psychological distress (PD) is prevalent and known to worsen outcomes amongst person living with Inflammatory bowel disease (IBD). Although management of disease related PD is now accepted as an integral part of treatment of IBD, barriers to accessing mental health services within the healthcare system persist. Mindfulness based interventions (MBI), particularly mindfulness-based stress reduction, (MBSR) have been shown to reduce PD and may have positive impacts on IBD through direct reductions in local and systemic inflammation. MBIs may be more accessible than psychotherapy. However, the evidence relating to the effectiveness of MBSR is varied and limited. This may be a result of how MBSR is implemented within the IBD population.

**Aims:**

To describe the implementation effectiveness of mindfulness-based interventions (MBIs) in adults living with inflammatory bowel disease.

**Methods:**

A systematic review of the literature was performed as follows: CINAHL, Cochrane Library, Embase, MEDLINE, PsycArticles, PsycInfo, Pubmed were systematically searched from inception to Feb 20th, 2025., Randomized controlled trials and cross-sectional studies were considered for inclusion. Abstracts, commentaries, protocols and reviews were excluded. Sources were screened by 2 reviewers; conflicts were resolved by and data were extracted by reviewers. This systematic review followed the preferred reporting items for systematic reviews and meta-analyses (PRISMA) guidelines.

**Results:**

Thirteen studies were included (n = 40-1068). Mindfulness Interventions were delivered online or in person and varied in duration (ranging from 8- 16 weeks). Four studies included IBD patients who had anxious or and depressive symptoms. Inclusion criteria varied across studies with disease activity including patients with remission, mild or active disease. Program persistence rates ranged from 44%-100%. Teaching components of the courses included mindfulness-based practices (formal and informal meditation) and 5/13 incorporated cognitive behavioural exercises and stress management techniques with 10/13 included homework assignments. Most MBIs included sharing experiences in a group setting. Professionals administering the interventions included trained nurses, psychologists, and social workers. Attrition rates were highest in studies that enrolled IBD patients with active disease.

**Conclusions:**

Mindfulness-based interventions are administered to IBD patients with varying program designs and implementation strategies and duration of program. The heterogeneity of different program components and implementation strategy makes it difficult to draw conclusions about how these interventions should be implemented to minimize attrition and improve effectiveness. Future research should be performed to identify barriers to MBI participation and identify appropriate patient-centered adaptations.

**Funding Agencies:**

None

